# Systematic Evaluation of Three microRNA Profiling Platforms: Microarray, Beads Array, and Quantitative Real-Time PCR Array

**DOI:** 10.1371/journal.pone.0017167

**Published:** 2011-02-11

**Authors:** Bin Wang, Paul Howel, Skjalg Bruheim, Jingfang Ju, Laurie B. Owen, Oystein Fodstad, Yaguang Xi

**Affiliations:** 1 Mitchell Cancer Institute, University of South Alabama, Mobile, Alabama, United States of America; 2 Department of Mathematics and Statistics, University of South Alabama College of Arts and Sciences, Mobile, Alabama, United States of America; 3 Department of Tumor Biology, Institute for Cancer Research, The Norwegian Radium Hospital, Rikshospitalet University Hospital, Oslo, Norway; 4 Department of Pathology, Stony Brook University School of Medicine, Stony Brook, New York, United States of America; University of Birmingham, United Kingdom

## Abstract

**Background:**

A number of gene-profiling methodologies have been applied to microRNA research. The diversity of the platforms and analytical methods makes the comparison and integration of cross-platform microRNA profiling data challenging. In this study, we systematically analyze three representative microRNA profiling platforms: Locked Nucleic Acid (LNA) microarray, beads array, and TaqMan quantitative real-time PCR Low Density Array (TLDA).

**Methodology/Principal Findings:**

The microRNA profiles of 40 human osteosarcoma xenograft samples were generated by LNA array, beads array, and TLDA. Results show that each of the three platforms perform similarly regarding intra-platform reproducibility or reproducibility of data within one platform while LNA array and TLDA had the best inter-platform reproducibility or reproducibility of data across platforms. The endogenous controls/probes contained in each platform have been observed for their stability under different treatments/environments; those included in TLDA have the best performance with minimal coefficients of variation. Importantly, we identify that the proper selection of normalization methods is critical for improving the inter-platform reproducibility, which is evidenced by the application of two non-linear normalization methods (loess and quantile) that substantially elevated the sensitivity and specificity of the statistical data assessment.

**Conclusions:**

Each platform is relatively stable in terms of its own microRNA profiling intra-reproducibility; however, the inter-platform reproducibility among different platforms is low. More microRNA specific normalization methods are in demand for cross-platform microRNA microarray data integration and comparison, which will improve the reproducibility and consistency between platforms.

## Introduction

MicroRNAs (miRNA) are a set of small, single-stranded, non-coding RNA molecules that bind the complementary 3′-UTR sequence of their target mRNA, preventing translation and inducing mRNA degradation [1], [2]. In this manner, miRNAs are predicted to regulate the expression of more than 30% of all human genes and are known to play a key role in many biological processes, including development, cell growth, differentiation, apoptosis, and tumorigenesis [3], [4], [5], [6], [7], [8], [9]. Recent studies have demonstrated the value of miRNA expression patterns for diagnostic, prognostic, and therapeutic uses [10], [11], thus heightening interest in miRNAs' potential as biomarkers for tumor development, progression, and chemosensitivity. Many well-established molecular and biological methodologies including microarray, cloning, northern blotting, quantitative real-time-PCR (qRT-PCR), in situ hybridization (ISH), and next generation sequencing (NGS) are now being successfully utilized for miRNA research [12], [13], [14], [15], [16], [17]. However, some unique signatures of miRNAs, such as their small total number and short length, have created technical obstacles for direct application in various array platforms, leading to a need for developing novel methodologies designed to measure miRNA expression with high specificity and sensitivity. Here, we will systematically compare three representative platforms that use different carriers: glass slide LNA microarray, beads-based array, and TLDA quantitative real-time PCR array. Each has been broadly applied to miRNA profiling.

Microarray technology has been successfully applied in the field of genomic and biological research over the past decade, allowing for the simultaneous profiling of tens of thousands of genes [12], [18]. Briefly, cyanine dye-labeled cRNA/cDNA is hybridized to its complementary detection probe on an array carrier (e.g. glass, silicon or nylon) and emits fluorescence in the presence of a laser. The intensity of fluorescence, as caught and measured using a scanner administered by professional software, relates the abundance of bound genes. However, miRNA is much shorter than mRNA, which provides an important caveat for its use in classically mRNA-favored technologies. Since mature miRNA contains only 19-25 nucleotides and detective probes on the array carrier require the complementary pairing of at least ∼20 base pairs, the full-length sequence of each mature miRNA must inherently be included in the probes. The effect is a wide Tm range for the entire miRNA population, resulting in decreased binding efficacy or fluorescent distortion. The application of Locked Nucleic Acid (LNA)-modified oligonucleotide probes has overcome this obstacle by modifying the LNA contents in the probe, eliminating the diversity of Tm values for individual miRNA probes by enhancing binding affinity and by leading to the improvement of miRNA detection specificity and sensitivity [19]. LNA array is one of multiple successful examples of microarray technology adopted into miRNA study. Thus, we selected it to represent microarray technology in our analysis.

Bead-based hybridization carries an expectation of increased specificity over glass-based microarray [20]. Five-micron polystyrene beads, uniquely colored (up to 100 colors) and covered with oligonucleotide capture probes specific for a single miRNA, are hybridized to biotinylated miRNA in the liquid phase and then they are stained with streptavidin-phycoerythrin. A flow cytometer (Luminex 200) directs a single column of beads through the path of two lasers; one laser is used to identify the particular miRNA by its bead color, and the other is used to detect bound quantities of miRNA based on the presence of the reporter molecule, phycoerythrin. Bead-based arrays allow for the inclusion of many combinations of miRNA capture beads into a single pool, which are adjusted based on the interaction of bead-coupled probes, and provides greater flexibility over time as miRNA are discovered and corresponding beads are created. Indeed, beads-based miRNA detection is both feasible and attractive for its high speed, heightened accuracy, and relatively low cost [20].

Quantitative real-time RT-PCR (qRT-PCR) assay is a rapid and reproducible methodology with a broad dynamic range compared to Northern blot or conventional RT-PCR when assessing RNA expression [21]. It has been widely applied in miRNA research for years and recognized therein as a gold standard [22]. TaqMan is a relatively mature technology for the qRT-PCR application and has been adopted into miRNA research utilizing a stem-loop structure specific for binding mature miRNA [15]. The development of TaqMan technology has led to an innovative design of Low Density Arrays (TLDA), a medium-throughput method for real-time RT-PCR that uses 384-well microfluidics cards. A single TLDA card may assay up to 384 miRNAs. In theory, this technology provides a feasible platform combining miRNA discovery and validation.

In this study, we will profile miRNAs from a panel of osteosarcoma xenografts using LNA microarray, beads array, and TLDA, respectively. Systematic comparison and evaluation within (intra-) and across (inter-) platforms will be performed.

## Results

### MicroRNA profiling using three platforms

A total of 40 human osteosarcoma xenograft specimens were employed for this study, which included 10 samples for each chemotherapeutic treatment (Cisplatin, Doxorubicin, and Ifosfamide) plus 10 non-treated samples. Locked Nucleic Acid (LNA) miRNA array, beads array, and TaqMan Low Density Array (TLDA) cards profiled 560, 319, and 664 human miRNAs, respectively. TLDA shared 508 and 231 miRNAs with LNA array and beads array, respectively, and LNA array has 221 overlapped miRNAs with beads array. A total of 213 miRNAs were shared by three platforms, as illustrated in Figure S1.

### Signal quality and background noise comparisons

**Figure 1:**
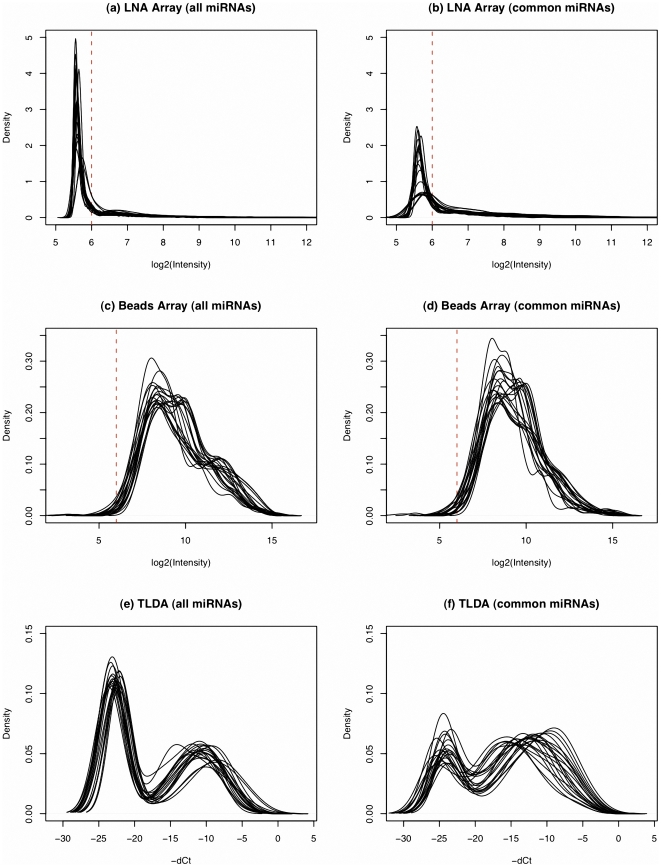
Expression distributions of miRNAs being profiled by the three platforms. Plots (a), (c) and (e) demonstrate the density curves of the expression of all miRNAs being profiled by LNA array, beads array, and TLDA, respectively. Plots (b), (d) and (f) compare the density curves of the expressions of the overlapped miRNAs from all three platforms. We took a log2 transformation to all intensity measures. For TLDA data, the 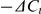 values were used.

Figure 1 compares the distributions of the log2 intensity measures for all the samples tested by the three platforms. The left panel illustrates the distribution of all miRNAs from three different platforms while the right panel displays the distribution of the 213 shared miRNAs. For each plot, we see that within each platform, the distributions of different profiles (without normalization) show similar patterns. By comparing each pair of plots in each row, especially (a) versus (b) and (e) versus (f), we find that the left modes are lower after the non-overlapped miRNAs are excluded. Meanwhile, the patterns of the distributions maintain similarity, indicating that a majority of the non-overlapped miRNAs are weakly expressed.

**Figure 2:**
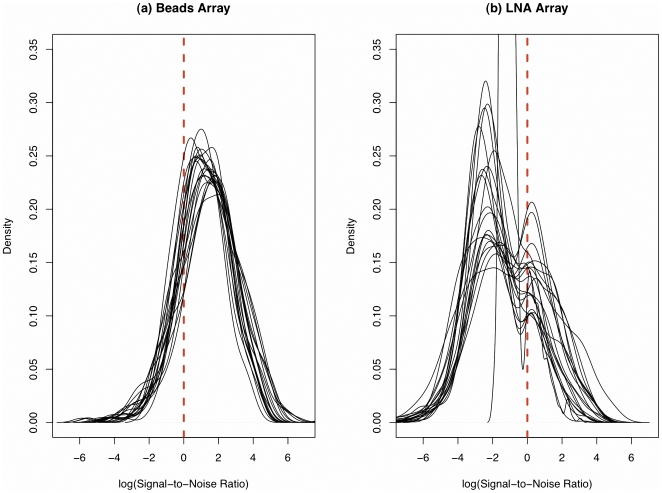
Signal-to-noise ratio comparison between beads array and LNA array. Plot (a) shows the density curves of the log signal-to-noise ratios for different samples tested by beads array, while the results for LNA array are demonstrated in plot (b). A log signal-to-noise ratio close to “one” indicates that the signal after background subtraction is close to the background noise.

The signal-to-noise ratio (SNR) is a statistical tool that measures the quality of the signals that are obtained from the arrays. When SNR is low, the background noise could dominate the measured expression signal and thus increase the uncertainty in evaluating gene expression levels. We computed the SNR for each miRNA in the LNA and beads arrays by dividing the background-subtracted signal by the estimated background noise. Results show that the beads array has an overall higher SNR than the LNA array (Figure 2). A number of probes have lower intensity than the background on the LNA array, which causes log (SNR) values to be negative. The SNR was not computed for TLDA because there were no estimates for the background noises in qRT-PCR.

### Intra-platform reproducibility

**Figure 3:**
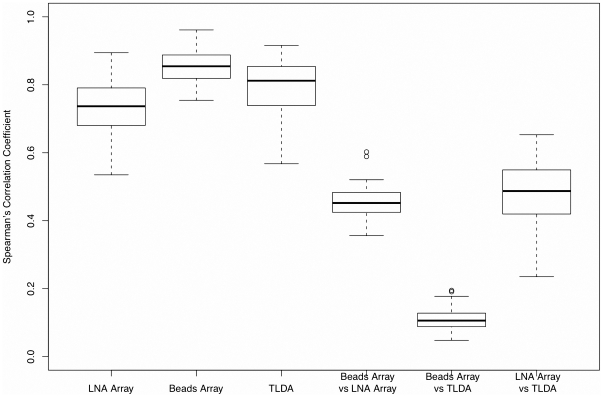
Intra- and inter-platform reproducibility comparisons. The first box plot to the left is based on Spearman's correlation coefficients between the LNA profiles of any two samples under the same treatment. The second and third plots are for the beads array and TLDA, respectively. The fourth plot is constructed based on the Spearman's correlation coefficients between the two profiles obtained by beads array and LNA array based on the same sample under the same treatment for all 10 samples. The fifth plot shows the results between beads array and TLDA, while the sixth plot shows the results between LNA array and TLDA.

To evaluate the intra-platform reproducibility, we calculated the rank-based Spearman's correlation coefficients among various miRNA profiles tested on different samples by the same platform. A stable platform is expected to produce similar results across different experiments. In other words, the results from the same sample using the same platform should be reproducible. The Pearson correlation coefficient analysis was banned because the study demonstrated that array profiling data were mostly non-linear [23], [24]. By using the Spearman's correlation coefficient measurement to evaluate intra-platform reproducibility, which adopts the rank information, the different scales used in each platform may be ignored and log-transformation can be avoided. From the three box plots on the left in Figure 3, we see that the beads array has the highest intra-platform reproducibility with a median Spearman correlation of 0.8544 and a standard deviation of 0.0475. The first and third quartiles are 0.8189 and 0.8877, respectively. TLDA has a median coefficient of 0.8118 with a standard deviation of 0.0745. The median coefficient of the LNA array is 0.7367, and the standard deviation is 0.0759. The correlation coefficients are computed based on the profiles of 213 overlapping miRNAs that are undergoing the same treatment. Our results demonstrate that the intra-platform reproducibility of all three platforms are acceptable and have no significant differences.

### Inter-platform consistency

The Spearman's correlation coefficients were also employed to evaluate the inter-platform reproducibility between any two platforms. The three box plots on the right in Figure 3 show the coefficients among the three platforms. Both beads array and TLDA display relatively good inter-platform reproducibility with LNA array. The median coefficient between LNA and beads arrays is 0.4521 with a standard deviation of 0.0537, and the median coefficient between LNA array and TLDA is slightly improved (0.4872) but with a slightly larger standard deviation of 0.0962. The reproducibility between beads array and TLDA is much lower, with a median coefficient of 0.1060 and a standard deviation of 0.0391.

### The evaluation of endogenous controls/probes for each platform

Our models measured the miRNA profiles of 10 xenograft samples. In order to evaluate the stability of endogenous controls/probes contained in each array, we employed 30 chemo drug treated samples and 10 untreated control samples to constitute the research models. The details will be described in the Material and Methods.

**Figure 4:**
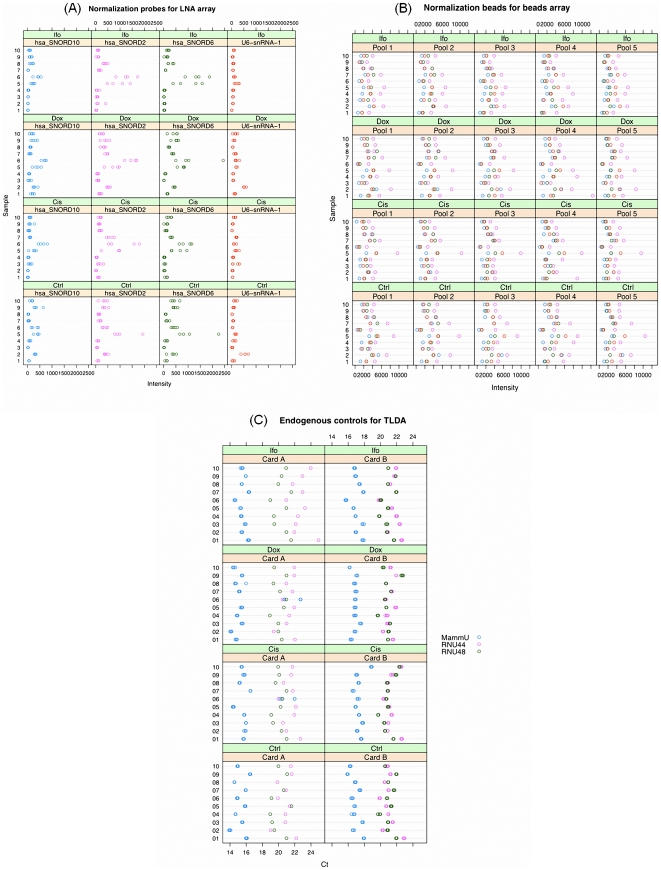
Comparison of three platforms' miRNA profiling data. **(A) LNA array**: The expressions of the four normalization probes in each LNA array are plotted, which have similar patterns across 10 samples under the four different treatments. **(B) Beads array**: The expressions of the four normalization beads in the five pools while testing 40 different samples are compared, which have similar patterns in the five pools across samples and indicate that these transcripts do not express significant variation between pools and samples. **(C) TLDA**: The profiles of the three endogenous controls commonly used by TLDA Cards A and B are demonstrated relatively stable across samples and treatments. Ctrl, Cis, Dox, and Ifo represent control, and three different chemo drug treatments.

LNA array includes 12 snoRNAs purposed for normalization. Table S1 compares their stability by computing coefficients of variation (CVs) based on the four replicates of 12 snoRNAs on each array. When processing raw data, if the signal intensity is less than 2 standard deviations from the background intensity, the probe will be flagged by the ImaGene 7.0 software. We found a few probes out of all 12 snoRNAs to be flagged across the 40 samples; however, hsa_SNORD2, hsa_SNORD3, hsa_SNORD6, hsa_SNORD10, and U6-snRNA-1 have relatively stable CVs compared to the remaining controls. In particular, hsa_SNORD2 has the smallest CVs and has no flagged probes across the 10 control samples. Because hsa_SNORD3 has a few outstanding measurements in some samples (data not show), we finally selected hsa_SNORD2, hsa_SNORD6, hsa_SNORD10, and U6-snRNA-1 for the further analyses. The expression patterns of these four controls, as determined by their absolute intensities, are presented in Figure 4A.

Beads array includes four normalization beads, which harbor probes that target ubiquitous small nucleolar RNAs (snoRNAs), useful for intra- and inter-sample normalization, upon successful identification of similarly expressed snoRNAs across multiple samples. Their relatively close expression patterns, determined by using the intensity values after background subtraction, are exhibited in Figure 4B.

A complete miRNA profile requires use of two TLDA cards, Cards A and B; Card A contains three endogenous controls (MammU6, RUN44, and RUN48) for relative quantitation, while B contains six endogenous controls (MammU6, RNU6B, RNU24, RNU43, RNU44, and RNU48). Figure 4C shows the profiles of the three endogenous controls common in the two cards (MammU6, RUN44, and RUN48) presented by Ct values.

**Figure 5:**
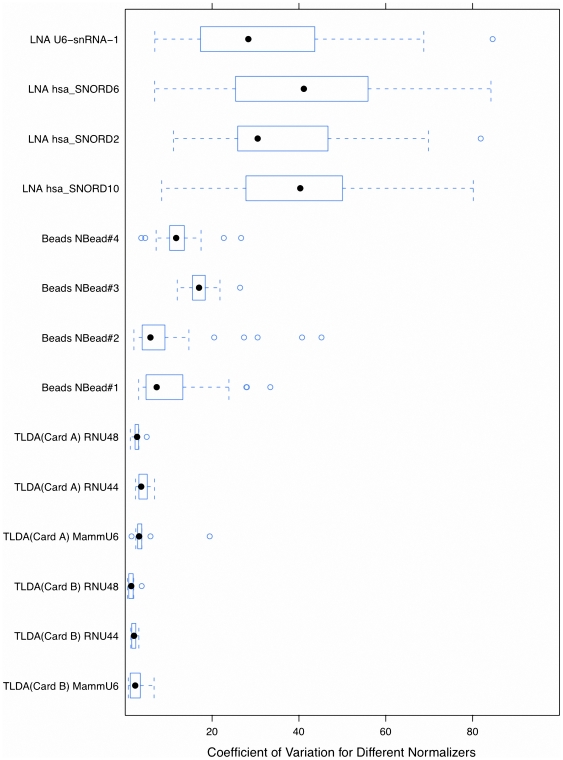
Stability evaluation for endogenous controls/probes from three platforms. The box plots of coefficient of variation (CV) values for each projected normalizer are calculated by using the measures of the replicates. Ctrl, Cis, Dox, and Ifo represent control, and three different chemo drug treatments.

To compare the stability of all endogenous controls/probes used within the three platforms, we again employed CVs by taking the expression levels into consideration (Figure 5). For the TLDA and LNA arrays, the CVs were computed based on the replicates of each control/probe in a single array, while in the beads array, the CVs were computed based on the intensities after intra-sample normalization for the four normalization beads from the five pools for each sample. From Figure 5, we find that the CVs of the four snoRNAs in LNA array have much larger means and variances compared to those on the other two platforms. Three endogenous controls in TLDA have the best performance with the small means and standard deviations.

### Proper normalization methods improve consistency between the platforms

The low data consistency using inter-platform comparison bans data integration across the platforms. There are many reasons that could lead to this concern, such as array design, properties, signal measurement, et al, but we assume that normalization might be one adjustable factor, as we previously reported [25]. In order to improve the inter-platform consistency, we introduced two additional and popularly used normalization methods [26], [27], quantile and loess, in parallel with using designated probes/beads for normalization. TLDA data was removed from the normalization by quantile and loess since its signals were presented as cycle threshold (CT), which denotes the number of PCR cycles required for the fluorescent signal to cross a designated threshold level above a calculated background, rather than being presented as intensity values for the probes, as LNA and beads array utilize. We demonstrated earlier that TLDA included the most stable endogenous controls, and, considering the suggestion of others [21], [22], we used the results from TLDA as a standard to evaluate the performance led by different normalization methods when applied to the analysis of LNA and beads arrays.

**Figure 6:**
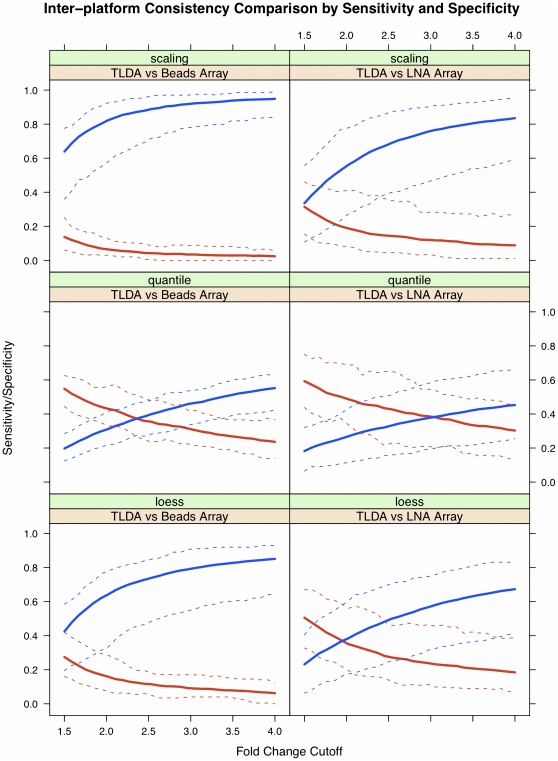
Inter-platform reproducibility measured by sensitivity and specificity. Sensitivities were compared to evaluate the consistency among the three platforms. The three plots in the first column compare the consistency between beads array and TLDA, the three plots in the second column compare the consistency between LNA array and TLDA, and the plots in the third column compare the consistency between beads array and LNA array. The results in the first, second and third rows are based on the profiles normalized using scaling by specific controls/probes, quantile normalization, and the cyclic loess method, respectively.

In this study, we also utilized sensitivity and specificity, two statistical measures of the performance of a binary classification, as test statistics of each platform to measure the inter-platform reproducibility. Our foremost assumption regarding the use of raw miRNA data for such a study is that a given miRNA from a single patient after a single treatment should maintain a consistent trend of up- or down-regulation across each platform, and an effective miRNA expression profiling platform should be capable of assessing the true direction of regulation. Thus, we compared the sensitivity and specificity of beads array (Figure 6; left panel) and LNA array (right panel), using the TLDA results as a reference, under different normalization methods with various fold-change cutoffs (x-axis). As shown in the left plot in the first row, the specificity is high when normalizing the profiling data of beads array by the four normalizers; however, the sensitivity is low. The loess method can improve the sensitivity and lower the specificity as a trade-off for the beads array data, while quantile normalization and the scaling method have similar effects on the beads data. By comparing the results of beads array with that of LNA array, we see that the consistency of LNA array in predicting the regulation trends (up- or down-regulated) is better than beads array, consistent with the results of inter-platform reproducibility as assessed by correlation coefficients. Additionally, quantile and loess normalization can improve the consistency of beads array and LNA array with TLDA, although the choice of optimal cutoff value could be slightly different.

## Discussion

MiRNA continues to attract more and more attention in the biomedical field due to its important roles in many cellular and molecular processes [3], [4], [5], [6], [7], [8], [9]; however, unlike DNA, mRNA, and protein, the genomic methodologies for miRNA discovery and validation are not well-developed when considering they are prospective in basic and clinical research. For example, there are relatively few specific normalization methods currently available for miRNA profiling analysis [25]. Even though many well-established methods for mRNA/cDNA and protein have been applied in miRNA analysis, their performance is still under evaluation, partially due to a shortage of effective validation methods that can assess the “real” presence and activity of miRNA. The total number of known human miRNAs to date is 939, according to the Sanger database, which is much lower than the number of known human gene transcripts (∼50 k). Any profiling normalization methods based on normal distribution can be applied to this small population estimation but the accuracy of those methods is questionable [25].

In this study, using a large cohort of samples constituting unique models exposed to different environments, we evaluated three representative platforms based on different mechanisms/carriers that have been applied for miRNA profiling, including LNA array, beads array, and TLDA. The distribution of intensity measures from each profile appear as variable patterns, which could be attributed to differences in signal collection and scales of measurement. Surprisingly, the SNR of the LNA array was secondary to the beads array; however, each probe for LNA had four technical duplicates, potentially overcoming the disadvantage of a weaker signal to some extent. Each array contained variable probe types based on the array's design or capacity, but 213 miRNAs were shared by three platforms. These miRNAs had similar distribution patterns in each array, indicating that they could represent each array for further analyses.

Git et al. compared microarray (from Agilent, Exiqon, Ambion, Invitrogen, and Combimatrix), qRT-PCR, and next generation sequencing (NGS) technologies for the miRNA profiles of three cell lines. They found that the actual overlap between the differentially expressed miRNAs was surprisingly low when comparing microarray, NGS, and qRT-PCR data [28]. Sato et al. also evaluated five commercial miRNA microarray platforms from Agilent, Exiqon, Ambion, Invitrogen, and Toray using two RNA samples and found the miRNA microarray to have high intra-platform repeatability and comparability to qRT-PCR but lower inter-platform concordance [29]. Using the designated normalization controls/probes for each array, we computed the intra- and inter-platform reproducibility. Our results demonstrated that the intra-platform reproducibility of all three platforms is reasonable, while the beads array is the most consistent. The LNA array and TLDA had the best inter-platform correlation while the TLDA and beads arrays were the least correlated. These observations are in agreement with the above reports [28], [29].

Multiple factors could potentially contribute to low inter-platform consistency, such as data collecting, mining, noise subtracting, etc. Normalization methods have thus been accepted to play an important role in data comparison and its integration across platforms [25], [26], [28], [30]. Specific controls have been recommended for miRNA normalization because data acquired from the small total number of miRNAs results in insufficient statistical power that can be used to adopt established mRNA profiling normalization methods [25]. The ideal controls should be consistently stable and highly abundant despite tissue types or treatments. Additionally, they should have properties similar to those of miRNAs, including size, biogenesis and stability [25]. Each platform employed herein includes a number of such controls with the potential to act as normalizers. Our unique models consisted of 10 untreated xenograft controls; each had three different chemo drug treatments, which resulted in a total of 40 samples. The beads array included four normalization beads of synthesized short oligos used to maintain consistency across samples and treatments. Similarly, the LNA array included 12 snoRNAs, and the TLDA contained three endogenous controls common to Cards A and B, which contributed to computing the relative quantity for each miRNA. When examining these controls, the beads array and TLDA performed better than the LNA array, which had only four controls that maintained relatively stable expression patterns across all samples.

We performed normalization for each array using the most stable controls. By adding two broadly applied normalization methods, quantile and loess, for comparison, we computed the sensitivity and specificity for each array. Endogenous controls in TLDA performed better across treatments, though its relative measures are calculated by 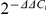, which is an unsuitable factor for normalization by the quantile and loess methods. Therefore, we used the TLDA data as a reference for evaluating the beads and LNA array because others have suggested qRT-PCR to be a gold standard for relative quantitation [22]. Our results indicate that proper normalization methods can improve the sensitivity and specificity of such platforms,; these results are in line with previous reports [26], [30].

Next generation sequencing (NGS) has been pushing for reform in the field of genomics [31] and has been projected to replace the use of microarray in the near future. Its introduction into miRNA research can be attributed to its ability to read short fragments in a high throughput pattern [32]. Most researchers, including us, agree with the prospective of this newly developed technology though it should be admitted that the NGS sequencing technology is still not a fully mature especially when compared to the microarray, which has more than twenty years of wide use [28]. In addition, NGS is based on RNA ligation, PCR amplification, and professional bioinformatic support, challenging its application in quantitative gene expression analysis due to cost, labor, and time consumption concerns [28]. Though it will take time for NGS to fully take over the microarray, its unique and outstanding sequence discovery capability is making NGS become a combatant to the microarray. Hopefully, the imminent advent of third generation sequencing provides further hope for more reliable and affordable genomic research platform availability for the future.

LNA array, beads array, and TLDA —three miRNA profiling platforms based on different mechanisms/carriers— exhibit better intra-platform consistency than inter-consistency, though each platform has its inherent merits and shortcomings. For example, LNA is an affordable platform with novel LNA technology and probe extending capacity but low SNR and unreliable normalization probes. The beads array has better intra-consistency but interference between short probes has to be considered. Even though the beads array has extending capacity, more input templates will reduce enthusiasm for its use. Both the LNA and beads arrays use a direct labeling process without template amplification, which eliminates the errors acquired from PCR. TLDA is an easy-to-use and effective solution for miRNA profiling but it is costly with limited extending capacity and no replicates are available at this time. Therefore, TLDA may be better applied to validation rather than discovery.

We are not advertising any platforms. We have attempted to evaluate the scientific merit of each applied miRNA profiling platform and provide useful evidence for our peers when selecting miRNA analytic strategies. Unfortunately, we could not identify any of these to be superior to the others due to their low inter-platform consistency. Thus, users should select a platform based on their available facilities, budget, interests, and/or loyalties; although, the selection of proper normalization and validation methods will clearly be one of the most critical factors in determining the best miRNA candidates. Therefore, we conclude that it is important to develop specific normalization methods for miRNA profiling in order to improve the accuracy of proofing data and provide the possibility of data integration across platforms, profound even in the future sequencing era.

## Materials and Methods

### Sample preparation and assessment of total RNA concentration, purity and quality

Forty human osterosarcoma xenografts were collected as described in the previous publication [33]. Frozen xenografts were ground by a stainless steel mortar and pestle under liquid nitrogen. Total RNA was isolated from the tissue powder using Trizol Reagent (Invitrogen, CA, USA) and was quantitated using a NanoDrop spectrophometer (Thermo Fisher Scientific Inc., MA, USA).

### MiRNA expression analysis using LNA array

Total RNA was labeled using the miRCURY LNA microRNA Array power labeling kit (Exiqon Inc., Denmark). All reagents used here were from Exiqon unless specifically mentioned. In brief, a 4 µl reaction volume consisting of 0.5 µl CIP buffer, 0.5 µl CIP enzyme, 1 µl Spike-in controls and 1 µg total RNA was incubated at 37°C for 30 min followed by 95°C for 5 min. The CIP reaction products were mixed with 3 µl labeling buffer, 1.5 µl Hy5 fluorescent label, 2 µl labeling enzyme, and 2 µl DMSO and were placed in the dark at 16°C for 60 min. The total volume of the labeled samples was adjusted to 200 µl by adding nuclease-free water, then mixed with an equal volume of 2× hybridization buffer at 95°C for 2 min and immediately cooled down on ice. The samples were loaded on the miRCURY LNA microRNA Array (Exiqon Inc.; based on miRbase 9.2) using a hybridization SureHyb chamber kit and gasket slide kit (Aglient Technologies, CA, USA). The slides were then rotated at 56°C for 16 hrs. After disassembling the chambers, the slides were washed in three steps. The first step was to immediately soak the slides in pre-warmed 2× salt buffer, containing 0.2% detergent solution, for 2 min. The second step was to rinse the slides with 1× salt buffer for 10 sec and then wash for 2 min in fresh 1× salt buffer. The third step was to use 0.2× salt buffer to wash the slides for 2 min. Finally, the slides were quick-dried by centrifuging at 1000 rpm for 2 min. Scanning was performed by an Axon GenePix Professional 4200A microarray scanner (Molecular Devices, CA, USA). Finally, the images were gridded and analyzed using ImaGene 7.0 software (BioDiscovery Inc., CA, USA).

### MiRNA expression analysis using beads array

Five µg (one µg per bead pool) of total RNA per sample in a 40 µl reaction volume were 3′-biotinylated using Luminex FlexmiR MicroRNA Labeling Kit (Luminex Corp., TX, USA), following manufacturer protocol. Using the Luminex FlexmiR MicroRNA Human Panel of reagents and xMAP beads, 8 µl of biotin-labeled total RNA, or water for the background control, were added to an equal volume of a single pool of microspheres (five pools total in the Human Panel targeting 319 miRNA), 14 µl of hybridization buffer and 20 µl water, mixed and covered to protect from evaporation and light, and denatured at 95°C for 3 min in a 96-well plate. Biotinylated miRNA were then hybridized to oligonucleotide-capture probes coupled to the carboxylated 5-micron polystyrene xMAP beads at 60°C for one hour. Following hybridization, the beads were washed and filtered, and 75 µl streptovidin-phycoerythrin:wash buffer (1∶300) reporter were added to the beads and incubated at room temperature on a plate shaker (600 rpm) for 30 min. Median fluorescence intensity (MFI) values were then measured using a Luminex 200 machine.

### MiRNA expression analysis using TLDA

The global profiling for miRNA expression for 40 samples was performed using the TaqMan Array Human MicroRNA Panel v2.0 (Applied Biosystems, CA, USA), which includes Cards A and B in a 384-well format. Card A contains 380 TaqMan MicroRNA Assays enabling the simultaneous quantitation of 377 human miRNAs plus 3 endogenous controls; Card B contains 293 assays for 287 human miRNAs plus 6 controls, which were experimental procedures following manufacturer instructions. In brief, total RNA was first reverse-transcribed with the Multiplex RT pool set (Applied Biosystems) through a reverse transcription (RT) step using the High-Capacity cDNA Archive Kit (Applied Biosystems), wherein a stem-loop RT primer specifically binds to its corresponding miRNA and initiates its reverse-transcription. The RT mix included 50 nM stem-loop RT primers, 1× RT buffer, 0.25 mM each of dNTPs, 10 U/µl MultiScribe reverse transcriptase, and 0.25 U/µl RNase inhibitor. The 7.5 µl reaction was then incubated for 30 min at 16°C, 30 min at 42°C, 5 min at 85°C, and then held at 4°C. The RT products were subsequently amplified with sequence-specific primers; we were using the Applied Biosystems 7900 HT Real-Time PCR system. Six µl of RT products were added to 444 µl nuclease free water and mixed with 450 µl TaqMan Universal Master Mix II, No UNG, then dispensed into the 384 wells by centrifugation. The reactions were incubated in a 384-well plate at 95°C for 10 min followed by 40 cycles of 95°C for 15 sec and at 60°C for 1 min. The data were collected and processed using the Plate Utility and Automation Controller software (Applied Biosystems). For each miRNA, the expression level was determined by 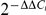 value calculation formula [34].

### Normalization methods

#### Intra-sample and inter-sample normalization for beads arrays

As recommended by the FlexmiR MicroRNA Human Panel Instruction Manual, intra- and inter-sample normalization is performed to normalize the median fluorescent intensity (MFI) across all the pools of a given sample or between two samples. To perform the intra-sample normalization, we first calculated the net MFI value for each result by subtracting the MFI values of the background. Second, we selected Pool 1 as the reference and computed the normalization factors by dividing the net MFI of all the normalization microspheres in the reference pool by the net MFI of the corresponding normalization microspheres from other pools. Third, we computed an intra-sample normalization factor for each pool by taking the median of the normalization factors of all the normalization microspheres across each pool. Lastly, we multiplied each calculated intra-sample normalization factor by all of the net MFI results within each associated pool.

After the intra-sample normalization, we performed the inter-sample normalization. First, we regarded the median net MFI value for each of the four normalization beads across the five pools as net MFI of the corresponding normalization microspheres. Second, we selected non-treated samples as controls and used the net MFI values to compute the normalization factors by dividing the net MFI of all of the normalization microspheres in the control sample by the net MFI of the corresponding normalization microspheres from treated samples. Third, we computed an inter-sample normalization factor for each sample by taking the median of the normalization factors of all the normalization microspheres across each sample. Lastly, we multiplied each calculated inter-sample normalization factor by all of the net MFI results within each associated sample.

#### Normalizing LNA array data by designated probes

Each LNA array contains 12 designated probes primarily designed for normalization purposes. Among them, U6-snRNA-1, hsa_SNORD2, hsa_SNORD6, and hsa_SNORD10 were chosen to normalize profiling data based on their proved stability earlier. For each selected probe, we took the median of the intensity measures after background subtraction from the four replicates for each array. Then the same inter-sample normalization algorithm for the beads arrays was applied to normalize the LNA arrays.

#### Computing coefficients of variation(CVs)

CV value is calculated by dividing the standard deviation of X by the mean value of X, then multiplying by 100 when defining X as a vector of the intensities of the replicates.

#### Cyclic loess

The cyclic loess method, sometimes known as the MA scatter plot, was first presented by Dudoit et al [35], [36]. Let Y be a profile for a sample under a treatment and X be a profile for the same sample as a control (without treatment). We first considered the M = log(Y/X) versus A = (log(Y)+log(X))/2 plot. Second, we fit a loess curve by regressing M on A and denote the fitted values by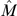. Third, we set D = exp ((M-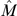)/2) and justified Y and X by Y' = Y*D and X' = X/D. The R loess function is used with the default smoothing parameter.

#### Quantile method

The quantile normalization method can deal with non-linear compressions by effectively taking the ranks of the observations into account and has been proposed by several authors [37], [38], [39]. It transforms all the replicates onto the same scale. Quantile normalization is applied to a matrix X of spotted intensities for all p genes and k replicates. Let 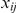 be the spot intensity for spot j on array i, 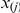 be a vector of jth smallest spot intensities across arrays, and 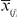 be the mean/median of 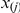. The vector 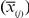 for 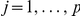 represents the “compromise” distribution. Let R be the matrix of row ranks associated with matrix X, then the quantile normalized value for spot j on array i is 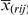. The quantile normalization method has been implemented in R package *affy* and is freely available from the *The Comprehensive R Archive Network* servers over the internet.

#### Computation of sensitivity and specificity

In this study, the sensitivity measures the proportion of actual positives that are correctly identified as such while specificity measures the proportion of negatives that are correctly identified.
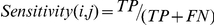


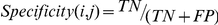
where TP (True Positive) is the number of up- or down-regulated miRNAs identified consistently by both the TLDA results and the platform being evaluated (LNA array or beads array), and TN (True Negative) is the number of non-differentially expressed miRNAs according to TLDA results and the platform being evaluated. FN (False Negative) refers to the number of miRNAs being classified as non-differentially expressed by the platform being evaluated while being classified as either up-regulated or down-regulated by the TLDA results. FP (False Negative) refers to the number of miRNAs being classified as either up-regulated or down-regulated by the platform being evaluated while being classified as non-differentially expressed by the TLDA results.

## Supporting Information

Figure S1 **Venn diagram illustrating the association of three miRNA profiling platforms.** LNA array, beads array, and TLDA profiled 560, 319, and 664 human miRNAs, respectively. TLDA shared 508 and 231 miRNAs with LNA array and beads array, and LNA array has 221 overlapped miRNAs with beads array. A total of 213 miRNAs were shared by three platforms.
(TIFF)

Table S1 **Stability evaluation of the designated probes on the LNA array.**
(DOC)
