# Supplementary figures and images for: Systematic Evaluation of Three microRNA Profiling Platforms: Microarray, Beads Array, and Quantitative Real-Time PCR Array
Source: PLoS One. 2011 Feb 11;6(2):e17167. doi: 10.1371/journal.pone.0017167 (PMC3037970; doi:10.1371/journal.pone.0017167)

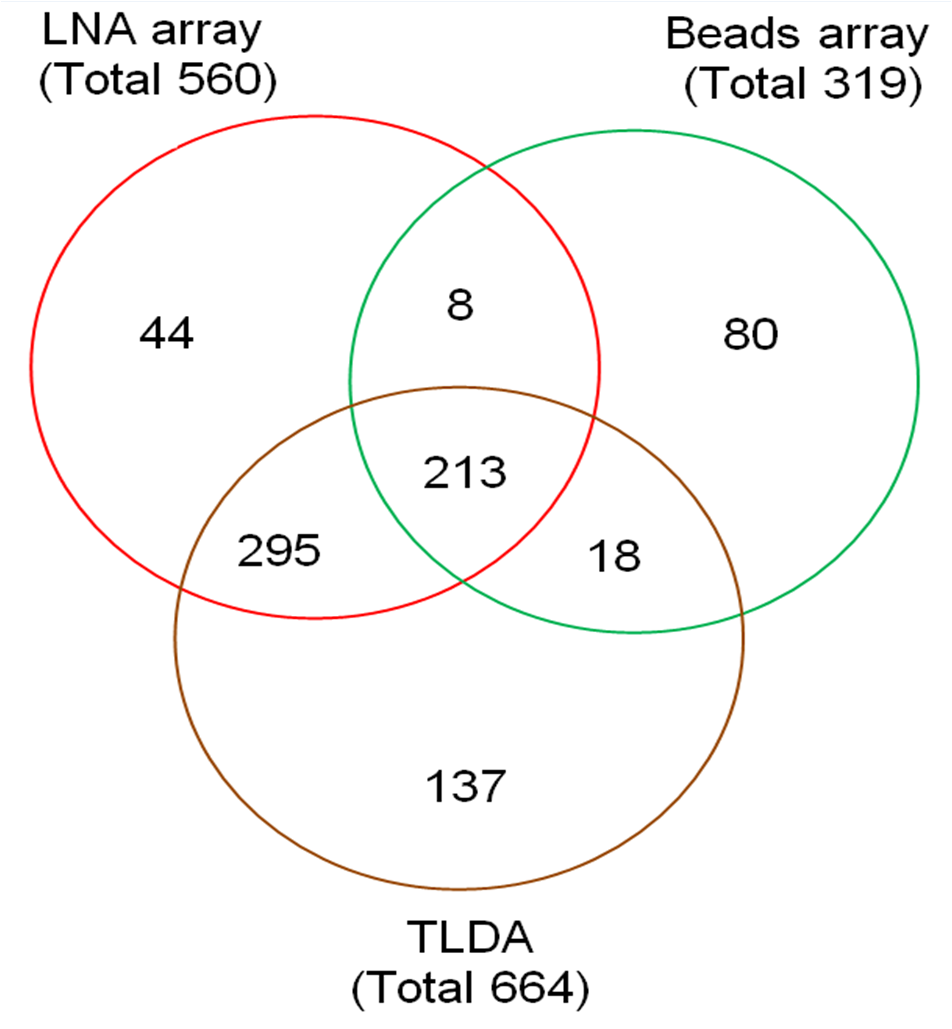

Supplement: Figure S1 — Venn diagram illustrating the association of three miRNA profiling platforms. LNA array, beads array, and TLDA profiled 560, 319, and 664 human miRNAs, respectively. TLDA shared 508 and 231 miRNAs with LNA array and beads array, and LNA array has 221 overlapped miRNAs with beads array. A total of 213 miRNAs were shared by three platforms. (TIFF) [file pone.0017167.s001.tif]
