# Supplementary material for: Systematic Evaluation of Three microRNA Profiling Platforms: Microarray, Beads Array, and Quantitative Real-Time PCR Array
Source: PLoS One. 2011 Feb 11;6(2):e17167. doi: 10.1371/journal.pone.0017167 (PMC3037970; doi:10.1371/journal.pone.0017167)
Supplement: Table S1 — Stability evaluation of the designated probes on the LNA array. (DOC) [file pone.0017167.s002.doc]

Table S1. Stability evaluation of the designated probes on the LNA array

| **Probes** | **Treatment** | **S-1** | **S-2** | **S-3** | **S-4** | **S-5** | **S-6** | **S-7** | **S-8** | **S-9** | **S-10** |
| --- | --- | --- | --- | --- | --- | --- | --- | --- | --- | --- | --- |
| **U6-snRNA-1** | Ctrl | 49.1* | 28.5* | - | 61.7 | 19.4 | - | 20.9 | - | 32.7 | 45.2 |
|  | Cis | - | - | 66.7* | 15.7* | 37.9 | 50.2 | 8.8 | - | - | 42.4 |
|  | Dox | 18.4 | 10 | 6.1* | 18.4 | 13.4 | 38.1 | 28.4 | 8.1* | 29.3 | 26.5* |
|  | Ifo | - | 9.2* | 7* | 18.9* | 44.9 | 29.1 | 5.5* | 26.3 | - | 24.9 |
| **U6-snRNA-2** | Ctrl | - | - | 45.1* | - | - | - | - | 9.8 | - | - |
|  | Cis | - | 39.1 | - | - | - | - | - | 31.5 | - | - |
|  | Dox | - | - | - | - | - | - | - | - | - | - |
|  | Ifo | 17.6* | 32* | - | 21.7 | - | - | 23.2* | - | - | 32.5* |
| **hsa_SNORD10** | Ctrl | 7.1* | 8.4 | - | 44.4 | 26 | 48.5 | - | - | 43.3 | 37.7 |
|  | Cis | - | - | 46.1* | - | 20.8* | 20.8* | 22.2 | - | 68.5* | 31.7 |
|  | Dox | 30 | 29.2 | 54.6* | 28.3* | 40.8 | 12.9 | 39.6 | 17.8 | 42.5 | 45 |
|  | Ifo | - | - | - | - | 40.4* | 34.3 | - | 45.6 | 45.8* | 40.2 |
| **hsa_SNORD118** | Ctrl | - | 12.9 | - | - | 35.4 | - | - | - | - | - |
|  | Cis | - | - | - | 60.7 | - | - | - | - | - | - |
|  | Dox | - | 27.1 | - | - | - | 43.9* | 49.3 | - | - | 6.8* |
|  | Ifo | - | - | - | - | - | 19.8* | - | - | - | 59.2 |
| **hsa_SNORD12** | Ctrl | - | - | - | - | - | - | - | - | - | - |
|  | Cis | - | - | - | - | - | - | - | - | - | - |
|  | Dox | - | - | - | - | - | - | - | - | - | - |
|  | Ifo | - | - | - | - | - | - | - | - | - | - |
| **hsa_SNORD13** | Ctrl | - | 7* | - | - | 63* | - | - | - | - | - |
|  | Cis | - | - | - | 19* | 24.5* | 13.8* | - | - | - | - |
|  | Dox | - | 38.5 | - | - | - | 62.9* | - | - | - | - |
|  | Ifo | - | - | - | - | - | 32.8* | - | - | - | - |
| **hsa_SNORD14B** | Ctrl | - | 18.7* | - | - | - | - | - | - | - | - |
|  | Cis | - | - | - | - | - | - | 10.8* | - | - | - |
|  | Dox | - | - | - | - | - | - | 17.1* | - | - | - |
|  | Ifo | - | - | - | - | - | - | - | - | - | - |
| **hsa_SNORD15A** | Ctrl | - | - | - | - | - | - | - | - | - | - |
|  | Cis | - | - | - | - | - | - | - | - | - | - |
|  | Dox | - | - | - | - | - | - | - | - | - | - |
|  | Ifo | - | - | - | - | - | - | - | - | - | - |
| **hsa_SNORD2** | Ctrl | 30.9 | 17.1 | 27.1 | 40.3 | 46.6 | 27 | 27.2 | 24 | 26.8 | 51.3 |
|  | Cis | 23.5 | - | 59.6 | - | 46.8 | 56.1 | 23.2 | 26 | 21.7 | 30.1 |
|  | Dox | 28.9 | 12.5 | 12* | 38.8 | 37.8 | - | 11.1 | 27.6 | 41.2 | 36.8 |
|  | Ifo | - | 114.3 | 15.1* | 75.7* | 56.7* | 28.7 | 27.2 | 21.9 | 10.3* | 25.8 |
| **hsa_SNORD3** | Ctrl | 41 | - | - | 30.1 | 43.6 | - | 54.5 | 32.3 | 46.6 | 44.1 |
|  | Cis | 40.4 | 96.6 | 78.3 | 53 | 33.4* | 29.7 | 32.4 | 16.5* | 22.9 | 27.1 |
|  | Dox | 12.1 | - | 26.4* | 17.6* | - | 34.5 | 61.9 | 8.4 | 29.9 | 75.8 |
|  | Ifo | - | 67.2* | 7.5 | 44.8 | - | 39.1 | 34.7 | 49.2 | 52.5 | 28.8 |
| **hsa_SNORD4A** | Ctrl | - | 36.1 | - | - | - | 14.2 | - | - | - | - |
|  | Cis | - | - | - | - | 26.5 | 31.8* | 12.9* | - | - | 7.3* |
|  | Dox | - | 38.7 | - | - | 23.1 | 9.6* | - | - | 14.6 | - |
|  | Ifo | - | - | - | - | 13.1 | 65.4* | - | - | - | - |
| **hsa_SNORD6** | Ctrl | 35.4 | 49.5 | - | 56.3 | 60.5 | 24.9 | 44 | 12.2 | 18.7 | 35.1 |
|  | Cis | 6.8* | - | - | - | 53.5 | 25.9 | 13.7 | 34.8 | 10.9* | 43.3* |
|  | Dox | 9.4 | 8.3 | - | 27.4 | 18 | 69.6 | 11.9* | 11 | 27.7 | 46.2 |
|  | Ifo | - | - | - | - | 55.5 | 29.6 | 6.8 | 41.4 | 18.2* | 28 |

Note: Ctrl,Cis,Dox, and Ifo represent the control, and three different chemo drug treatments. The values present within the table are coefficients of variation (CVs), which were calculated by using four replicates of each probe without flags across samples, while an asterisk will be given if the CV was computed based on the three un-flagged replicates. A dash means or there are more than two replicates were flagged or the data were missing. S-n(1-10) represents the individual sample.
